# Artificial Domestication Enhances Bioactive Profiles and Antioxidant Capacity in Two Wild Asteraceae Plants

**DOI:** 10.3390/plants14233662

**Published:** 2025-12-01

**Authors:** Aihong Zheng, Hanfeng Gao, Zhixin Wei, Dongyang Sun, Shuyu Han, Xuling Ren, Xuhua Wan, Yonggang Cao, Keshun Wu, Bo Sun

**Affiliations:** 1 Pingliang Agricultural Technology Promotion Station, Pingliang 744000, China; 15120405001@163.com (A.Z.); gaohanfenglz@163.com (H.G.); renxl1020@163.com (X.R.); 18215358180@163.com (X.W.); cyg9024@126.com (Y.C.); 18293380613@163.com (K.W.); 2 Key Laboratory of Agricultural Integrated Pest Management, Academy of Agriculture and Forestry Science, in Qinghai Province, Qinghai University, Xining 810016, China; 3 College of Horticulture, Sichuan Agricultural University, Chengdu 611130, China; 202201704@stu.sicau.edu.cn (Z.W.); 2024305096@stu.sicau.edu.cn (D.S.); 2024305095@stu.sicau.edu.cn (S.H.)

**Keywords:** *Taraxacum mongolicum*, *Sonchus oleraceus*, artificial domestication, bioactive compounds, antioxidant capacity, Asteraceae, wild vegetables

## Abstract

*Taraxacum mongolicum* Hand.-Mazz. and *Sonchus oleraceus* L. are perennial herbaceous species with strong antioxidant capacities; however, these species have not been widely utilized as medicinal materials because their natural populations are limited, and manual harvesting is labor-intensive. In this study, we compared the yield, bioactive components (in leaves and roots), and antioxidant capacity of domesticated and wild populations of both species. Domesticated cultivation significantly increased the contents of ascorbic acid, total phenols, and flavonoids in *T. mongolicum* leaves, total phenols in *T. mongolicum* roots, and total phenols and flavonoids in *S. oleraceus* leaves. Domestication markedly enhanced antioxidant capacity (with the exception of *S. oleraceus* roots) and the free radical scavenging capacity in the leaves and roots of both wild vegetable species (with the exception of *S. oleraceus* roots). Domesticated cultivation also substantially improved yield, with *T. mongolicum* yield increasing by 12,850 kg/ha (fresh weight) and *S. oleraceus* yield increasing by 18,600 kg/ha (fresh weight). Correlation analysis showed that the free radical scavenging capacity of leaves in both species was significantly negatively correlated with the soluble sugar content, whereas the soluble sugar content in roots was significantly positively correlated with the soluble protein content. Overall, our findings will aid further studies of bioactive substances and antioxidant-related genes in *T. mongolicum* and *S. oleraceus.*

## 1. Introduction

Wild vegetables have attracted increased attention because they are generally less contaminated by pesticides and chemical fertilizers, are rich in trace elements, and have diverse edible parts and culinary uses [1]. However, wild vegetables are often patchily distributed and characterized by unstable yield and quality. Natural populations alone are thus often insufficient for meeting market demand, and large-scale, unregulated collection may adversely affect ecosystems and biodiversity. This has substantially increased the market potential of artificially domesticated and cultivated wild vegetables. Selecting superior varieties from numerous wild species and promoting their industrial-scale cultivation represents an important direction for the development of the wild vegetable industry.

Asteraceae (the aster family) is an important group of plant resources in China [2]. Its characteristic capitulum (head) inflorescence and diverse reproductive strategies confer strong environmental adaptability [3]. Species such as *Taraxacum mongolicum* Hand.-Mazz., *Sonchus oleraceus* L., *Arctium lappa* L., and *Smallanthus sonchifolius* (Poepp.) H. Rob. are common wild vegetables of the Asteraceae family in China. *T. mongolicum* and *S. oleraceus* are especially prevalent in the Longdong region of Gansu Province. Owing to their strong adaptability and health-promoting properties, they are widely consumed locally.

*T. mongolicum* is a common wild vegetable, and the entire plant can be consumed. In traditional Chinese medicine, it is considered cold in nature and bitter–sweet in taste, and is believed to strengthen the liver and gallbladder, clear heat and toxins, promote diuresis, reduce swelling, and lower cholesterol [4]. The whole plant contains various nutritious components, including taraxol, taraxacin, choline, organic acids, and inulin [5]. Previous studies have shown that polysaccharides from *T. mongolicum* can inhibit inflammatory responses [6]; other studies indicate that *T. mongolicum* has significant anti-tumor effects [7]. As a food-medicine homologous species with strong adaptability, a wide distribution, and high nutritional value, *T. mongolicum* is a promising plant resource. The tender leaves and underground tender roots are the main edible parts of *S. oleraceus*. This species is rich in vitamins (including vitamins A, B1, and B2) and other nutrients such as calcium, iron, protein, and fat [8,9]. It has been reported to clear heat and toxins, clear the lung, relieve cough, cool the blood, drain dampness, resist oxidation, promote growth and development, and help prevent and treat anemia [10,11,12,13]. Previous studies have also shown that *S. oleraceus* can kill certain insect pests and inhibit the growth of some bacteria [14].

To date, research on *T. mongolicum* and *S. oleraceus* has mainly focused on their medicinal value [15,16,17,18]. Some researchers have examined the effects of different light intensities, light qualities, and wavelengths on the growth, photosynthetic pigments, and chicoric acid accumulation of *S. oleraceus* under in vitro culture conditions [19]. Other studies have explored antioxidant components, nutrients, and volatile substances in wild *S. oleraceus* [20,21]. Liu et al. identified flavonoid compounds in crude extracts of *T. mongolicum* before and after fermentation and verified their antioxidant capacity [22]. Additional work has also confirmed the presence of antioxidant constituents in *T. mongolicum* [23]. However, few studies have examined the changes in how artificial domestication and cultivation affect the nutritional quality and antioxidant components in different plant parts of these two Asteraceae wild vegetables.

Here, we used wild and domesticated cultivar materials of *T. mongolicum* and *S. oleraceus* to systematically clarify changes in their nutritional value and antioxidant components following artificial domestication and cultivation. We analyzed differences in the contents of bioactive substances and antioxidant capacity among different plant parts after domestication and cultivation. The results showed that domesticated cultivation significantly increased the contents of ascorbic acid, total phenols, and flavonoids in *T. mongolicum* leaves, total phenols in *T. mongolicum* roots, and total phenols and flavonoids in *S. oleraceus* leaves. Domestication markedly enhanced antioxidant capacity (with the exception of *S. oleraceus* roots) and the free radical scavenging capacity in the leaves and roots of both wild vegetable species (with the exception of *S. oleraceus* roots). Domesticated cultivation also substantially improved the yield of two Asteraceae wild vegetables. Overall, our aim was to generate data to promote the development and utilization of *T. mongolicum* and *S. oleraceus*.

## 2. Results

### 2.1. Phenotypic Analysis of Wild Plants and Domesticated Plants

**Figure 1:**
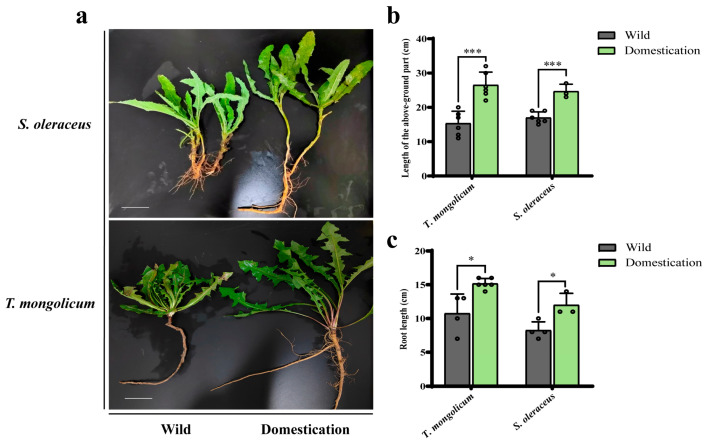
Plant morphology and traits of wild vs domesticated *Taraxacum mongolicum* and *Sonchus oleraceus*. (**a**) Plant morphology; (**b**) Length of the above-groud part; (**c**) Root length. *, **, *** indicate significant difference at 0.05, 0.01 and 0.001 level, ns = not significant, respectively. “
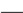
”, this scale shows a length of 5 cm.

There were pronounced phenotypic differences in plant traits between wild and domesticated/cultivated *T. mongolicum* and *S. oleraceus* (Figure 1a). Both the aboveground length and root length of domesticated *T. mongolicum* and *S. oleraceus* were markedly greater than those of wild plants (Figure 1b,c). Leaf length and leaf width were significantly larger in domesticated *T. mongolicum* than in wild plants (Table 1). In addition, the aboveground parts and roots of domesticated plants were enlarged, with both aboveground fresh weight and root fresh weight significantly exceeding those of wild plants (Figure S1C,F). The yield of domesticated *T. mongolicum* reached 51,870 kg/ha (fresh weight), which was 12,850 kg/ha higher than that of the wild population (with two harvests per year). The yield of *S. oleraceus* reached 128,160 kg/ha (fresh weight), 18,600 kg/ha higher than that of the wild population. Under an adequate water and fertilizer supply, one crop of *S. oleraceus* (harvesting only young leaves) can be obtained every 20–30 days, allowing up to six harvests per year (Table 1).

### 2.2. Analysis of Photosynthetic Pigments in Different Parts of Wild Plants and Domesticated Plants

**Figure 2:**
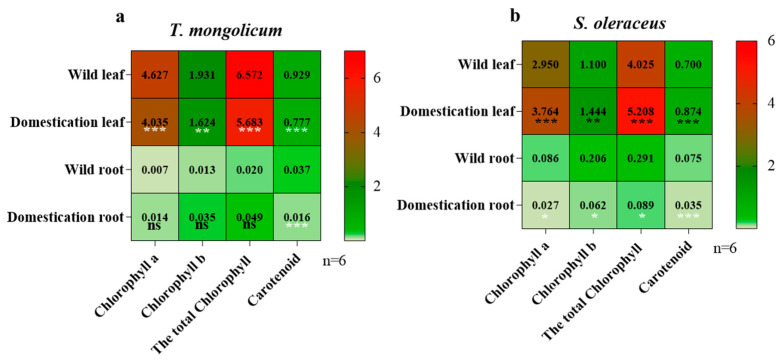
Photosynthetic pigments of wild vs domesticated *Taraxacum mongolicum* (**a**) and *Sonchus oleraceus* (**b**). *, **, *** indicate significant difference at 0.05, 0.01 and 0.001 level, respectively. The color of the significance marker (*) is adjusted based on the shade of the background color, solely for enhancing visibility, and does not indicate differences in significance levels.

To assess differences in photosynthetic pigments between domesticated and wild plants, we quantified the chlorophyll a, chlorophyll b, total chlorophyll, and carotenoid contents. The chlorophyll a, chlorophyll b, total chlorophyll, and carotenoid contents of leaves were reduced in domesticated plants compared with wild plants; in roots, no significant differences in the chlorophyll a, chlorophyll b, and total chlorophyll content were observedin the roots, while the carotenoids, but the carotenoid content was markedly lower in domesticated plants than in wild plants (Figure 2a). In *S. oleraceus*, the chlorophyll a, chlorophyll b, total chlorophyll, and carotenoid contents were higher in leaves in domesticated plants than in wild plants; in roots, the chlorophyll a, chlorophyll b, total chlorophyll, and carotenoid contents were significantly lower in domesticated plants (Figure 2b). The chlorophyll a, chlorophyll b, total chlorophyll, and carotenoid contents in both *T. mongolicum* and *S. oleraceus* were higher in leaves than in roots (Figure 2a,b). The contents of chlorophyll a, chlorophyll b, total chlorophyll, and carotenoids were higher in *T. mongolicum* than in *S. oleraceus*, with respective values of 2.17 mg/g vs. 1.71 mg/g, 0.90 mg/g vs. 0.70 mg/g, 3.08 mg/g vs. 2.40 mg/g, and 0.44 mg/g vs. 0.42 mg/g (Figure 2a,b).

### 2.3. Analysis of Soluble Sugars and Soluble Proteins in Different Parts of Wild Plants and Artificially Domesticated Plants

**Figure 3:**
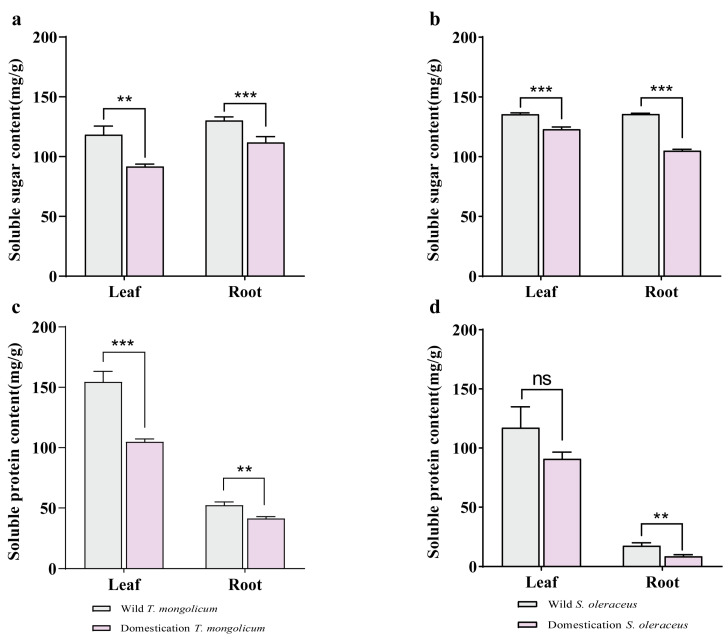
The content of soluble sugar and protein of leaf and root in two kinds of species. (**a**) The content of soluble sugar of leaf and root in *T. mongolicum*; (**b**) The content of soluble sugar of leaf and root in *S. oleraceus*; (**c**) The content of soluble protein of leaf and root in *T. mongolicum*; (**d**) The content of soluble protein of leaf and root in *S. oleraceus*; *, **, *** indicate significant difference at 0.05, 0.01 and 0.001 level, ns = not significant, respectively.

To examine differences in stress resistance between domesticated and wild plants, we quantified the soluble sugar and soluble protein contents in both groups. The soluble sugar and soluble protein contents were significantly lower in both the leaves and roots of domesticated *T. mongolicum* compared with wild plants (Figure 3a,c). In *S. oleraceus*, domestication significantly reduced the soluble sugar content in both leaves and roots; the soluble protein content in leaves did not differ significantly between domesticated and wild plants, whereas the soluble protein content in the roots of domesticated plants was significantly decreased (Figure 3b,d).

In *T. mongolicum*, the soluble sugar content was lower in leaves (105.09 mg/g) than in roots (121.14 mg/g), and the soluble protein content was higher in leaves (129.75 mg/g) than in roots (46.98 mg/g) (Figure 3a,c). In *S. oleraceus*, both the soluble sugar and soluble protein contents were higher in leaves than in roots (129.36 mg/g vs. 120.38 mg/g; 140.06 mg/g vs. 12.99 mg/g). *T. mongolicum* had a lower soluble sugar content than *S. oleraceus* (113.12 mg/g vs. 124.87 mg/g), whereas its soluble protein content was higher (88.37 mg/g vs. 58.53 mg/g).

### 2.4. Antioxidant Components in Different Parts of Wild Plants and Domesticated Plants

**Figure 4:**
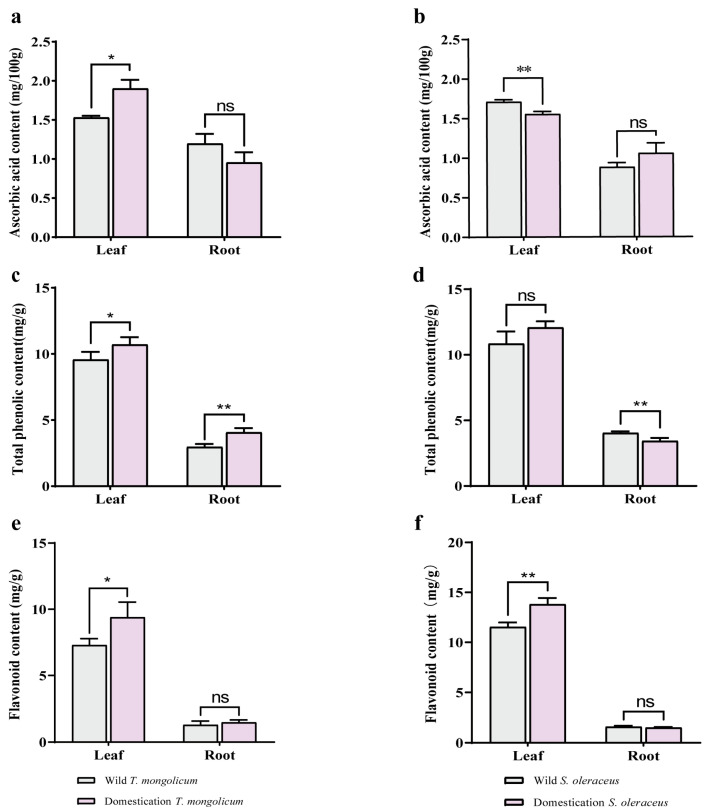
The content of antioxidant components of the leaf and root in two kinds of species. (**a**) The content of ascorbic acid of leaf and root in *T. mongolicum*; (**b**) The content of ascorbic acid of leaf and root in *S. oleraceus*; (**c**) The content of total phenolic of leaf and root in *T. mongolicum*; (**d**) The content of total phenolic of leaf and root in *S. oleraceus*; (**e**) The content of flavonoid of leaf and root in *T. mongolicum*; (**f**) The content of flavonoid of leaf and root in *S. oleraceus*; *, **, *** indicate significant difference at 0.05, 0.01 and 0.001 level, ns = not significant, respectively.

To characterize differences in antioxidant components between domesticated and wild plants, we quantified ascorbic acid, total phenols, and flavonoids in both groups. The contents of ascorbic acid, total phenols, and flavonoids in leaves were higher in domesticated *T. mongolicum* compared with wild plants; the ascorbic acid and flavonoid contents in roots did not differ significantly between domesticated and wild plants, whereas the total phenol content was significantly higher in domesticated roots than in wild roots (Figure 4a,c,e). In *S. oleraceus*, domestication significantly decreased the ascorbic acid content in leaves but increased the total phenol and flavonoid contents; in roots, the ascorbic acid and flavonoid contents did not differ significantly between domesticated and wild plants, while the total phenol content in domesticated roots was noticeably reduced (Figure 4b,d,f). The contents of ascorbic acid, total phenols, and flavonoids were higher in leaves than in roots for both *T. mongolicum* and *S. oleraceus* (Figure 4). The ascorbic acid content in *T. mongolicum* was similar to that in *S. oleraceus* (average values of 1.40 mg/100 g and 1.30 mg/100 g, respectively); the total phenol content was higher in *S. oleraceus* than in *T. mongolicum* (7.60 mg/g vs. 6.83 mg/g); and the flavonoid content was higher in *S. oleraceus* than in *T. mongolicum* (7.10 mg/g vs. 4.86 mg/g) (Figure 4).

### 2.5. Antioxidant Capacity in Different Parts of Wild Plants and Domesticated Plants

**Figure 5:**
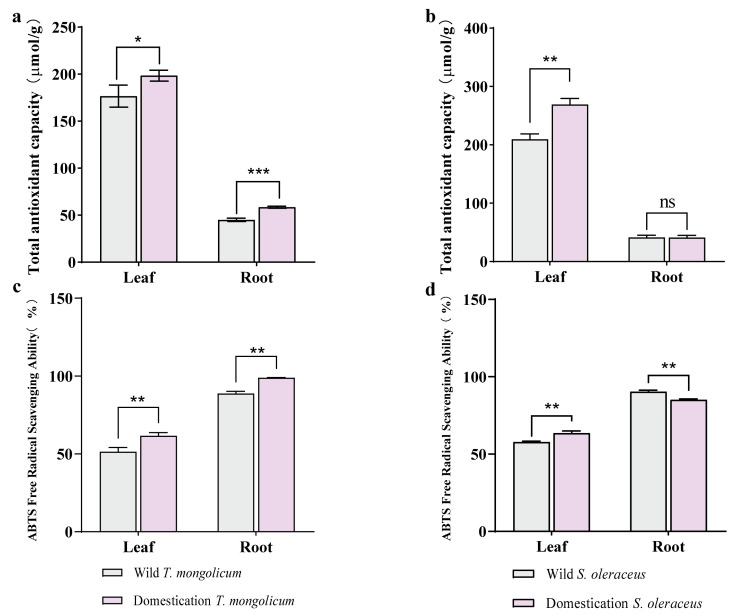
Antioxidant capacity of leaves and roots in two kinds of species. (**a**) The total antioxidant capacity of leaf and root in *T. mongolicum*; (**b**) The total antioxidant capacity of leaf and root in *S. oleraceus*; (**c**) The ABTS free radical scavenging ability of leaf and root in *T. mongolicum*; (**d**) The ABTS free radical scavenging ability of leaf and root in *S. oleraceus*; *, **, *** indicate significant difference at 0.05, 0.01 and 0.001 level, ns = not significant, respectively.

The total antioxidant capacity of wild and domesticated plants was evaluated using the FRAP assay. The antioxidant capacity was markedly increased in both the leaves and roots of domesticated *T. mongolicum* compared with wild plants (Figure 5a). In *S. oleraceus*, domestication significantly enhanced the antioxidant capacity in leaves, and no significant difference was observed in the root antioxidant capacity between domesticated and wild plants (Figure 5b). The antioxidant capacity of leaves was greater than that of roots in both *T. mongolicum* and *S. oleraceus* (Figure 5a,b). The total antioxidant capacity of *S. oleraceus* was higher than that of *T. mongolicum* (140.3 µmol/g vs. 119.36 µmol/g) (Figure 5a,b).

The free radical scavenging capacity of wild and domesticated plants was assessed using the ABTS^+^ assay. The free radical scavenging capacity in both leaves and roots was significantly higher in domesticated *T. mongolicum* relative to wild plants (Figure 5c). In *S. oleraceus*, the free radical scavenging capacity of leaves was enhanced in domesticated plants compared with wild plants, whereas the scavenging capacity of roots was significantly reduced (Figure 5d). Roots had a higher free radical scavenging capacity than leaves in both *T. mongolicum* and *S. oleraceus* (Figure 5c,d). The free radical scavenging capacities of *T. mongolicum* and *S. oleraceus* were similar, with average values of 75.23% and 74.23%, respectively (Figure 5c,d).

### 2.6. Principal Component Analysis of Components in Different Parts of Wild Plants and Artificially Domesticated Plants

**Figure 6:**
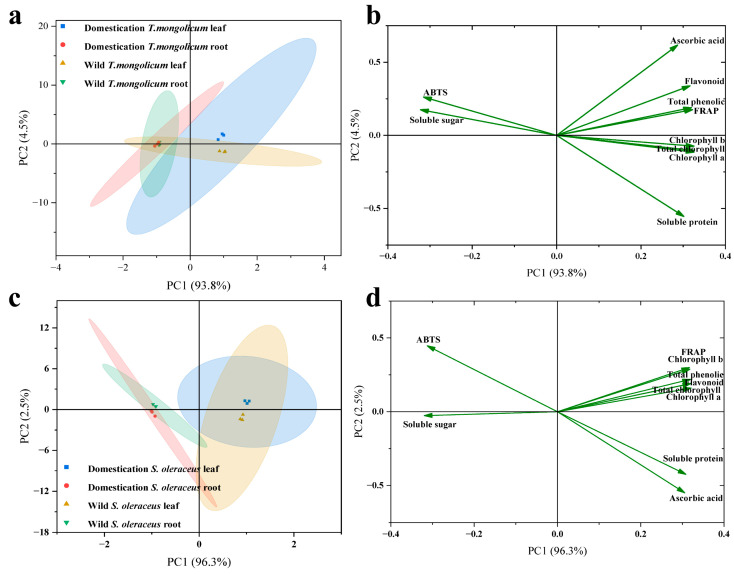
Principal component analysis of ten components in leaves and roots of two vegetables. (**a**) The principal component analysis (PCA) of leaf and root in *T. mongolicum*; (**b**) The loading plot of PCA of ten components leaf and root in *S. oleraceus*; (**c**) The PCA of leaf and root in *T. mongolicum*; (**d**) The loading plot of PCA of ten components leaf and root in *S. oleraceus*.

To more intuitively characterize differences in nutrient components and antioxidant capacity between the leaves and roots of wild and domesticated plants, we performed principal component analysis (PCA). In *T. mongolicum*, the first principal component (PC1) accounted for 93.8% of the total variance and the second principal component (PC2) for 4.5%, together explaining 98.3% of the variability in the original data. Domesticated leaves and roots were clearly separated in the score plot, indicating pronounced differences between them along these two principal components. The loading plot showed that soluble protein and ascorbic acid contributed most strongly to PC1 and PC2, and that soluble protein was negatively correlated with ABTS^+^ antioxidant capacity (Figure 6a,b).

In *S. oleraceus*, PC1 accounted for 96.3% and PC2 for 2.5% of the total variance, together explaining 98.8% of the original data variability. Domesticated leaves and roots were also clearly separated, indicating substantial differences between organs in terms of the two principal components. The loading plot revealed that soluble protein, ascorbic acid, and ABTS^+^ antioxidant capacity contributed most to these components. Soluble protein was positively correlated with ascorbic acid, whereas both soluble protein and ascorbic acid were negatively correlated with ABTS^+^ antioxidant capacity (Figure 6c,d).

### 2.7. Correlation Analysis of Components in Different Parts of Wild Plants and Artificially Domesticated Plants

**Figure 7:**
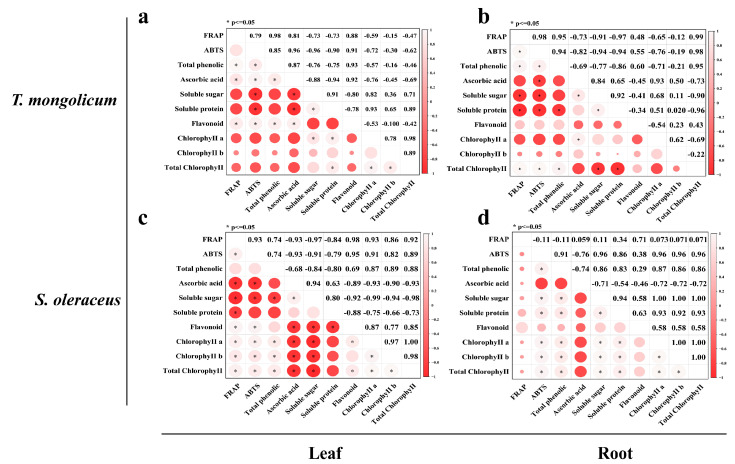
Correlation analysis of ten components in leaves and roots of two vegetables. (**a**) Correlation analysis of ten components in leaf of *T. mongolicum*; (**b**) Correlation analysis of ten components in root of *T. mongolicum*; (**c**) Correlation analysis of ten components in leaf of *S. oleraceus*; (**d**) Correlation analysis of ten components in root of *S. oleraceus*.

We assessed correlations among 10 components in the leaves and roots of *T. mongolicum* and *S. oleraceus*. In *T. mongolicum* leaves, correlation coefficients ranged from −0.96 to 0.98. ABTS^+^ was strongly negatively correlated with both soluble sugar and soluble protein, and ascorbic acid was also significantly negatively correlated with soluble sugar and soluble protein (*p* ≤ 0.05). In contrast, soluble protein was significantly positively correlated with soluble sugar (*p* ≤ 0.05) (Figure 7a). In *T. mongolicum* roots, correlation coefficients ranged from −0.97 to 0.99. FRAP was significantly negatively correlated with soluble protein and significantly positively correlated with total chlorophyll (*p* ≤ 0.05), whereas ascorbic acid was significantly positively correlated with soluble sugar (*p* ≤ 0.05) (Figure 7b).

In *S. oleraceus* leaves, correlation coefficients ranged from −0.99 to 1.00. ABTS^+^ was significantly negatively correlated with both soluble sugar and ascorbic acid, and ascorbic acid was significantly positively correlated with soluble sugar (*p* ≤ 0.05) (Figure 7c). In *S. oleraceus* roots, correlation coefficients ranged from −0.76 to 1.00. ABTS^+^ was negatively correlated with ascorbic acid, but significantly positively correlated with chlorophyll b and total chlorophyll (*p* ≤ 0.05) (Figure 7d).

## 3. Discussion

Numerous studies have shown that environmental conditions, cultivation practices, and other factors strongly influence the accumulation of bioactive substances in plants [24,25,26]. In this study, we artificially domesticated and cultivated two wild Asteraceae vegetables and compared their nutrient profiles, including chlorophyll, carotenoids, soluble sugar, soluble protein, ascorbic acid, total phenols, flavonoids, and antioxidant capacity, between species, between wild and domesticated varieties, and between different organs. After artificial domestication and cultivation, the yield of both wild vegetables significantly increased; the contents of ascorbic acid, total phenols, and flavonoids increased; the antioxidant capacity improved; and the contents of soluble sugar and soluble protein (stress-related components) significantly decreased. Correlation analyses further revealed that the free radical scavenging capacity of leaves in both species was markedly negatively correlated with the soluble sugar content, whereas the soluble sugar content in roots was positively correlated with the soluble protein content. The contents of chlorophyll, carotenoids, soluble sugar, soluble protein, ascorbic acid, total phenols, flavonoids, and antioxidant capacity were all higher in leaves than in roots (the free radical scavenging capacity was all higher in roots than in leaves), with the exception of the soluble sugar content in *T. mongolicum* leaves, which was lower than that in roots. Compared with *S. oleraceus*, *T. mongolicum* had higher contents of chlorophyll, carotenoids, and soluble protein and lower soluble sugar, total phenol, flavonoid contents, and antioxidant capacity; the ascorbic acid content and free radical scavenging capacity were similar in these two species. Overall, these data will aid the industrial cultivation of these two wild vegetables and the subsequent exploration of genes involved in the regulation of bioactive substances and antioxidant capacity.

Soluble sugars and soluble proteins are major forms of carbohydrate metabolism and transient storage, and they play key roles in regulating plant stress resistance [27,28]. Wang et al. reported that tetraploid non-heading Chinese cabbage had higher soluble sugar contents (19.78–50.86 mg/g), whereas diploid non-heading Chinese cabbage had higher soluble protein contents (5.06–47.03 mg/g); differences in individual nutritional indices and overall nutritional quality between diploid and tetraploid types varied with genotype and specific nutritional traits [29]. Hu et al. preliminarily evaluated the cold resistance of eight *Pennisetum* forage varieties under natural low-temperature stress and found that the Bond 1 hybrid *Pennisetum* accumulated high levels of soluble sugars and soluble proteins (8–10 mg/g and 35.7–64.22 mg/g, respectively) and showed strong cold resistance, suggesting that soluble sugars and soluble proteins may play a positive role in plant tolerance to low temperatures [30]. In our study, we found that *T. mongolicum* and *S. oleraceus* contained substantial amounts of soluble sugar and soluble protein. Notably, soluble sugar accumulated at high levels in both the roots and leaves of the two species, and the accumulation of soluble protein was high, particularly in leaves. This may be related to species-specific characteristics, growth environment, and stress conditions. The soluble sugar and soluble protein contents of both species decreased after domestication and cultivation. This reduction may be attributed to changes in growth conditions associated with artificial domestication, especially altered soil nutrient status, which could have reduced the accumulation of soluble sugars and soluble proteins. These findings are generally consistent with the conclusions of previous studies [31,32].

Components such as ascorbic acid, flavonoids, total phenols, carotenoids, and chlorophyll are key indicators for evaluating the nutritional value and quality of vegetables. In this study, artificial domestication and cultivation markedly increased the contents of ascorbic acid, flavonoids, and total phenols in *T. mongolicum*, as well as the contents of total phenols, flavonoids, total chlorophyll, and carotenoids in *S. oleraceus* leaves. In both species, the contents of ascorbic acid, flavonoids, and total phenols were higher in leaves than in roots, and the measured values were generally consistent with those reported previously [33,34]. Jiang et al. reported that 60% light transmittance favored the accumulation of chlorophyll and total phenols in *T. mongolicum*, whereas 80% light transmittance was more conducive to flavonoid accumulation [35]. Cao et al. found that, under a moderate water supply for 15 days, the accumulation of ascorbic acid, total phenols, total flavonoids, and other substances in *S. oleraceus* leaves was enhanced [36]. In our study, although the nutritional quality of the two vegetables was significantly improved compared with their wild counterparts, a gap was observed between the measured indices and the maximum values reported in previous studies. Thus, in future work, the nutritional quality of these two vegetables could be further enhanced by optimizing cultivation practices.

Previous research using liquid chromatography–mass spectrometry (LC–MS) indicates that the total phenolic content in ethanol extracts of the aerial parts of *Solidago graminifolia* L. Salisb. can reach 192.69 mg/g, which is 25 times higher than that detected in *S. oleraceus* (average of 7.60 mg/g in the whole plant in our study) and 28 times higher than that in *T. mongolicum* (average of 6.83 mg/g in the whole plant in our study). In addition, the hydroxycinnamic acid content in *S. graminifolia* is approximately 9.98 mg/g [37]. Studzińska-Sroka et al. determined that the whole-plant total phenolic content of *Galinsoga parviflora* was 98.3 mg/g and the chlorogenic acid content was 2.0 mg/g using ultra-high-performance liquid chromatography (UPLC) [38]. These findings indicate clear differences in the total phenolic content among Asteraceae species and suggest that the total phenols in *T. mongolicum* and *S. oleraceus* may also include small amounts of hydroxycinnamic acids.

Total antioxidant capacity is an important indicator for assessing both the quantity and activity of antioxidants in a sample. Shen et al. evaluated the antioxidant capacity of five pickled and dried mustard brands using the ABTS^+^, ORAC, and FRAP methods [39]. Zhang et al. measured the antioxidant capacity of different parts of blueberry using the DPPH, ABTS^+^, and FRAP methods and found that the trends obtained with these assays were consistent [40]. Guo et al. also reported significant differences in antioxidant capacity among cauliflowers of different colors, indicating that antioxidant levels in plants vary among varieties [41]. In our study, the overall trends in antioxidant capacity measured by the two methods were consistent, with domesticated plants generally exhibiting significantly higher antioxidant capacity than wild plants (except for the roots of *S. oleraceus*). However, total antioxidant capacity measured by the FRAP method in different plant parts (wild and domesticated) of the two species was higher in leaves than in roots, whereas the ABTS^+^ free radical scavenging capacity measured by the ABTS^+^ assay was higher in roots than in leaves. This discrepancy may stem from the presence of multiple antioxidant components in the two species, which may exert different inhibitory or reducing effects depending on the reaction system.

Sanja Vojvodić et al. measured the total antioxidant capacity of various Asteraceae plants using the FRAP assay and reported values ranging from 19.82 to 774.43 μmol/g, which is broadly consistent with our results (119.46 μmol/g for whole *T. mongolicum* plants and 140.3 µmol/g for whole *S. oleraceus* plants) [42]. Dai et al. found that the ABTS^+^ radical scavenging capacity of *Blumea balsamifera* (Asteraceae) increased with the concentration of its methanol extract, reaching a maximum of 99.87%. In our study, the ABTS^+^ radical scavenging capacity of different parts of *T. mongolicum* and *S. oleraceus* (both before and after artificial domestication) ranged from 51% to 99%, which is consistent with these previous findings [43].

## 4. Materials and Methods

### 4.1. Cultivation and Domestication of Wild Plants

The experimental site was located in Caofeng Town, Kongtong District, Pingliang City, Gansu Province, China (35.567253° N, 106.902931° E). The area is a tableland at an elevation of 1340 m, with an annual mean temperature of 8–10 °C, annual sunshine duration of 2136–2376 h, annual precipitation of 500–695 mm, and a frost-free period of 165–190 days, representing a typical dry-farming, rain-fed region on the Loess Plateau. The wild vegetables were domesticated and cultivated in open fields from April 2022 to June 2025. Robust wild plants were selected (in Caofeng Town, Kongtong District, Pingliang City, Gansu Province, China (35.567253° N, 106.902931° E), and seedlings with 3–4 leaves and intact roots were excavated (with roots kept as intact as possible and with an appropriate amount of soil attached); they were then transplanted into pre-prepared plots for domestication and cultivation. The domestication site soil was loessial, with a pH of 7.6–8.3 and organic matter content of 0.6–0.8%; the temperature during the growing period was 15–28 °C, with natural light for 8–10 h per day. Adequate basal fertilizer was applied before soil preparation (15,000 kg/ha farmyard manure + 600 kg/ha NPK compound fertilizer). Planting furrows were opened and irrigated once before transplanting, followed by a second irrigation 2 days after transplanting, with each irrigation amounting to 60% of field capacity. The plant spacing for field planting was 10 cm × 20 cm, and no chemical pesticides were used. After five consecutive generations of domestication and propagation (selection criteria for each generation: plant height ≥ 15 cm, absence of obvious diseases and insect pests, and number of leaves ≥ 5 to ensure population uniformity), the F5 progeny were used for field production. During the domestication period, 150 kg/ha of urea and 67.5 kg/ha of potassium dihydrogen phosphate were topdressed after each harvest to promote regrowth of the next crop.

### 4.2. Sample Collection and Growth Indicators

Samples (at the fresh-eating stage) were collected at 7:00 a.m. with soil attached and transported back to the laboratory. Samples for physiological and biochemical analyses were then freeze-dried using a freeze dryer. A ruler was used to measure the aboveground length and root length of both vegetables, while an electronic balance was used to determine aboveground and root fresh weights. An electronic scale was employed to measure yield.

### 4.3. Chlorophyll and Carotenoid Content Determination

Referring to the method of Sun et al. [44], an appropriate amount of sample was weighed, and 96% (*v*/*v*) ethanol was added for extraction by grinding in an ice bath. After thorough homogenization, the mixture was transferred to a volumetric flask and brought to a fixed volume. Following centrifugation, the supernatant was collected, and the absorbance was measured at 665, 649, and 470 nm. The contents of chlorophyll and carotenoids were then calculated.

### 4.4. Soluble Sugar Content Determination

Following the methods of Clegg [45] and Sun et al. [44], the clarified supernatant was collected. Next, 1 mL of supernatant was mixed with 0.5 mL of anthrone–ethyl acetate and then 5 mL of concentrated sulfuric acid. After incubation in a 90 °C water bath for 5 min, the mixture was rapidly cooled in ice water. Once it reached room temperature (25 °C), the absorbance was measured at 630 nm, and the total soluble sugar content was calculated using a sucrose standard curve.

### 4.5. Soluble Protein Content Determination

Following the methods of Bradford [46] and Sun et al. [44], 1 mL of supernatant was mixed sequentially with 1 mL of distilled water and 10 mL of 0.01% Coomassie Brilliant Blue G-250 staining solution. After thorough mixing to ensure complete reaction, the mixture was incubated in the dark for 5 min. The absorbance was then measured at 595 nm. A standard curve was constructed using bovine serum albumin (BSA) as the standard, and the soluble protein content of the samples was calculated using the corresponding linear regression equation.

### 4.6. Ascorbic Acid Content Determination

Following the method of Sun et al. [44], a Waters Spherisorb C18 chromatographic column (250 × 4.6 mm i.d.; 5 µm particle size; Waters Corporation, Milford, MA, USA) was used, with 0.1% oxalic acid as the mobile phase for isocratic elution at a flow rate of 1.0 mL·min^−1^. Using authentic ascorbic acid as the standard, the total ascorbic acid content was calculated from the absorbance at 243 nm.

### 4.7. Total Phenolic Content Determination

Following the methods described by Folin et al. [47] and Sun et al. [44], the ethanol extract was centrifuged, and 300 μL of the supernatant was mixed with 1.5 mL of 0.2 mol·L^−1^ Folin–Ciocalteu reagent. After incubation under dark conditions for 24 h, an appropriate amount of saturated sodium carbonate solution was added. The mixture was then kept at room temperature for 20 min, after which the absorbance was measured at 760 nm using a spectrophotometer, with gallic acid as the standard.

### 4.8. Flavonoid Content Determination

The method of Pirie et al. [48] was used with slight modifications. An appropriate amount of sample was weighed into a centrifuge tube, and 3 mL of extraction solution was added. The mixture was kept at room temperature (25 °C) for 24 h and then centrifuged, and the supernatant was collected as the flavonoid extract. Subsequently, 300 µL of the flavonoid extract was mixed with 900 µL of 95% ethanol, 60 µL of 2% aluminum chloride solution, 60 µL of 1 mol·L^−1^ potassium acetate solution, and 1.68 mL of distilled water. After thorough mixing, the reaction was allowed to proceed at room temperature for 40 min, and the absorbance was measured at 415 nm using a spectrophotometer. A standard curve was constructed using quercetin as the standard, and the flavonoid content of the samples was then calculated.

### 4.9. FRAP Determination

Following the method of Benzie et al. [49], the FRAP working reagent was prepared, and an appropriate amount was added to the extracted sample, which was then incubated at 37 °C for 10 min. The absorbance was subsequently measured at 593 nm using a spectrophotometer, and the FRAP value was calculated from an FeSO_4_·7H_2_O standard curve and expressed as μmol·g^−1^ dry weight (DW).

### 4.10. ABTS^+^ Determination

Following the method of Re et al. [50], an appropriate amount of ammonium persulfate was added to an ABTS^+^ solution to generate ABTS^+^ free radicals, and the mixture was kept in the dark for 16 h. The absorbance of the ABTS^+^ solution was then adjusted to 0.700 (±0.020) at 734 nm using acetate buffer (pH 4.5). Subsequently, 300 μL of extract was added to 3 mL of the ABTS^+^ solution, and after standing at room temperature for 2 h, the absorbance was measured at 734 nm.

### 4.11. Data Analysis and Processing

Excel 2019 (Version 16.0) was used for data processing, and two-tailed *t*-tests were applied for significance analysis. GraphPad Prism (Version 8.0.2.263), OriginPro 2024b (Version 10.1.5.132), and Adobe Illustrator CC (Version 23.0.2) were used to generate figures.

## 5. Conclusions

Interest in healthy living has led to a steady increase in demand for wild vegetables. Selecting superior species from numerous naturally occurring wild vegetables for industrial-scale cultivation is a key direction for the development of the wild vegetable industry. In this study, we analyzed differences in yield, bioactive components, and antioxidant capacity between domesticated and wild varieties of two species under artificial domestication and cultivation. The results showed that domesticated cultivation significantly increased the yield of the edible parts of both wild vegetables. Leaves and roots of both species were rich in bioactive substances, including ascorbic acid, carotenoids, flavonoids, and total phenols, and exhibited strong antioxidant capacity, indicating their potential for the development of functional foods. Thus, the artificial cultivation and commercialization of these wild plants can ensure a continuous and stable supply without substantially compromising their natural quality. This approach is critically important for protecting the ecological environment and preventing the depletion of plant resources in natural habitats.

## Figures and Tables

**Table 1:** Phenotypic differences between domesticated and wild species of two vegetables.

Varieties	Leaf Height (cm)	Leaf Width (cm)	Yield (kg/ha)
Wild *T. mongolicum*	10.86	2.06	39,020
Domestication *T. mongolicum*	21.11 **	3.65 *	51,870 **
Wild *S. oleraceus*	10.45	1.63	109,560
Domestication *S. oleraceus*	12.18	2.05	128,160 **

* and ** indicate significant and extremely significant differences at 0.05 and 0.01 levels, respectively.

## Data Availability

Data are contained within the article and Supplementary Materials.

## Supplementary Materials

The following supporting information can be downloaded at: https://www.mdpi.com/article/10.3390/plants14233662/s1, Figure S1: Detailed phenotypes of leaves and roots in two kinds of wild and domesticated vegetables.

## References

[1] Huang K., Su S.L. (2017). Ethnobotanical study on wild edible plants in diet culture of Zhuang Nationality in Western Guangxi. J. Plant Resour. Environ..

[2] Lang L.J., Cui S.J., Zhu Q.J., Yang L., Wang Y., Jiang B., Xiao C.J. (2022). Study on in vitro Lipid-Lowering Activity of 33 Compositae Plants from West Yunnan. J. Dali Univ..

[3] Barreda V.D., Palazzesi L., Tellería M.C., Olivero E.B., Raine J.I., Forest F. (2015). Early evolution of the angiosperm clade Asteraceae in the Cretaceous of Antarctica. Proc. Natl. Acad. Sci. USA.

[4] Qiao Y.J., Zhang Y., Wu S.H., Liu H.R., Deng G.H. (2023). Processing technology of *T. mongolicum* tea. Shanghai Agric. Sci. Technol..

[5] Li W., Lee C., Kim Y.H., Ma J.Y., Shim S.H. (2017). Chemical constituents of the aerial part of *Taraxacum mongolicum* and their chemotaxonomic significance. Nat. Prod. Res..

[6] Wu Z.G., Cheng S.F., Li Q.T., Huang K., Zhang Y.B. (2023). Research progress on extraction, isolation and biological activities of plant polysaccharides. Light Ind. ST.

[7] Yang C., Yan Q.Z., Tang J., Xia B.H., Lin L.M., He Q.Z., Liao D.F. (2018). Study on chemical composition of volatile oil from Taraxaci Herba and its antiinflammatory and anti-tumor activities. China J. Tradit. Chin. Med. Pharm..

[8] Fioroto M.A., Toniazzo T., Giuntini E.B., Oliveira P.V., Purgatto E. (2024). Mineral nutrients and protein composition of non-conventional food plants (*Pereskia aculeata* Miller, *Sonchus oleraceus* L. and *Xanthosoma sagittifolium* (L.) Schott). J. Food Compos. Anal..

[9] Karkanis A., Ntatsi G., Vasilakakou E., Karavidas I., Ntanasi T., Rumbos C.I., Athanassiou C.G. (2024). Combining *Tenebrio molitor* frass with inorganic nitrogen fertilizer to improve soil properties, growth parameters, and nutrient content of *Sonchus oleraceus* crop. Bioresour. Technol..

[10] Laabar A., Kabach I., Asri S.E., Kchikich A., Drioua S., Hamri A.E., Faouzi M.E.A. (2025). Investigation of antioxidant, antidiabetic, and antiglycation properties of *Sonchus oleraceus* and *Lobularia maritima*(L.) Desv. extracts from Taza, Morocco. Food Chem. Adv..

[11] Abdelhameed M.F., El-Baset M.A., Khattab A.R., Taher R.F., El-Saied M.A., Elkarim A.S.A., Essa A.F., El-Rashedy A.A., Farag M.A., Imagawa H. (2025). Hepatoprotective action of *Sonchus oleraceus* against paracetamol-induced toxicity via Nrf2/KEAP-1/HO-1 pathway in relation to its metabolite fingerprint and in silico studies. PLoS ONE.

[12] Guo A.H., Ren J.Y., Wang P., Duan H., Yang Q., Hou M.J., Gao P. (2016). Antimicrobial and antioxidant activities of different parts from *Sonchus oleraceus*. Guizhou Agric. Sci..

[13] Idan A.H., Al-nayili A., Saady N.M.C. (2025). Green synthesis of single phase of ZrO_2_ nanoparticles via *Sonchus asper* aqueous extract: Antibacterial, antioxidant, cytotoxic, and photocatalytic applications. Clean Technol. Environ. Policy.

[14] Zulkefle N.N., Zainal N., Wahyuni D.K., Mahmood S., Ramarao K.D.R., Chin K.L. (2025). Antimicrobial properties of *Sonchus* species: A review. Asian Pac. J. Trop. Biomed..

[15] Aissani F., Grara N., Guelmamene R. (2022). Phytochemical screening and toxicity investigation of hydro-methanolic and aqueous extracts from aerial parts of *Sonchus oleraceus* L. in Swiss albino mice. Comp. Clin. Pathol..

[16] Vecchia C.A.D., Locateli G., Serpa P.Z., Gomes D.B., Ernetti J., Miorando D., Zanatta M.E.D.C., Nunes R.K.S., Wildner S.M., Gutierrez M.V. (2022). *Sonchus oleraceus* L. promotes gastroprotection in rodents via antioxidant, anti-inflammatory, and antisecretory activities. Evid. Based Complement. Altern. Med..

[17] Chen L., Lin M., Wang Y.Y., Wang X.S., Qi C.C., Fan R.Y., Su S.L., Duan J.L., Liu F., Guo S. (2025). *Taraxacum mongolicum* total triterpenoids and taraxasterol ameliorate benign prostatic hyperplasia by inhibiting androgen levels, inflammatory responses, and epithelial-mesenchymal transition via the TGFβ1/Smad signalling pathway. J. Ethnopharmacol..

[18] Li X.R., Guo Y., Deng X.X., Jiao Y.N., Hao H.F., Dong Q.Q., Sun H., Han S.Y. (2025). *Taraxacum mongolicum* Hand.-Mazz extract disrupts the interaction between triple-negative breast cancer cells and tumor-associated macrophages by inhibiting RAC2/NF-κB p65/p38 MAPK pathway. J. Ethnopharmacol..

[19] Leite J.J.F., De Assis R.M.A., Rocha J.P.M., Cossa M.C.V., De Oliveira T., Coelho A.D., Da Silva A.C.B., Mendonca S.C., Bertolucci S.K.V., Pinto J.E.B. (2025). Sow thistle (*Sonchus oleraceus* L.) propagation in vitro: Wavelength, photon flux density and natural ventilation effects on its growth and chicoric acid content. S. Afr. J. Bot..

[20] Gendy A.E.G.E., Mohamed N.A., Sarker T.C., Hassan E.M., Garaa A.H., Elshamy A.I., Abd-ElGawad A.M. (2024). Chemical composition, antioxidant, and cytotoxic activity of essential oils in the above-ground parts of *Sonchus oleraceus* L.. Plants.

[21] Botella Á.M., Hellín P., Hernández V., Dabauza M., Robledo A., Sánchez A., Fenoll J., Flores P. (2024). Chemical composition of wild collected and cultivated edible plants (*Sonchus oleraceus* L. and *Sonchus tenerrimus* L.). Plants.

[22] Liu N., Song M., Wang N.F., Wang Y., Wang R.F., An X.P., Qi J.W. (2020). The effects of solid-state fermentation on the content, composition and in vitro antioxidant activity of flavonoids from dandelion. PLoS ONE.

[23] Cao X., Li G.Q., Xie J.Y., Wu M.Q., Wang W.H., Xiao L., Qian Z.M. (2024). Screening antioxidant components in different parts of dandelion using online gradient pressure liquid extraction coupled with high-performance liquid chromatography antioxidant analysis system and molecular simulations. Molecules.

[24] Wang C.Y., Zhou X., Guo D., Zhao J.H., Yan L., Feng G.Z., Gao Q., Yu H., Zhao L.P. (2019). Soil pH is the primary factor driving the distribution and function of microorganisms in farmland soils in northeastern China. Ann. Microbiol..

[25] Chen L.Y., Xu H., Xu Z.G., Xiao J. (2021). Effects of different habitats on the growth, chlorophyll content and chlorophyll fluorescence characteristics of medicinal and edible plants *Sambucus chinensis* Lind. Ecol. Sci..

[26] Li D.D., Liang Z.S., Pubu Z.M., Yang Z.Q., Han R.L., Xu X.X. (2020). Flavonoids content and flavonoids synthetic key enzyme activities in Alfalfa under drought stress. Acta Bot. Boreal.-Occident. Sin..

[27] Zhu Z., Jiang J.Y., Jiang C.J., Li W. (2011). Effects of low temperature stress on SOD activity, soluble protein content and soluble sugar content in Camellia sinensis leaves. J. Anhui Agric. Univ..

[28] Cheng T.L., Li H.Y., Wu H.W., Liu Z.X., Wu X., Yang S., Zhang H.X., Yang X.Y. (2015). Comparison on osmotica accumulation of different salt-tolerant plants under salt stress. For. Res..

[29] Wang J., Jiang J.Q., Yang Y., Zhou H.Z., Li Y., Hou X.L., Liu T.K. (2025). Comparative analysis of nutritional quality of diploid and autotetraploid of 17 non-heading Chinese cabbage. Acta Agric. Shanghai.

[30] Hu Y.B., Liu Y.N., Zhang J., Liang X.Y., Ji Y. (2025). Preliminary evaluation on cold tolerance of 8 Pennisetum forage varieties (lines) under natural low temperature. J. Grassl. Forage Sci..

[31] Lv L. (2022). Effects of Pig Manure Biogas Slurry Irrigation on Soil Quality and Growth of Four Vegetable Crops. Master’s Thesis.

[32] Sun C.Q., Yang Y.J., Guo Z.L., Qu F. (2015). Effects of fertilization and density on soluble sugar and protein and nitrate reductase of hybrid foxtail millet. J. Plant Nutr. Fertil..

[33] Guo S.P., Huang Y., Wang W.J., Huang Y.P. (2014). Difference comparison of antioxidant activity among different varieties of Amaranth and Dandelion. Shandong Agric. Sci..

[34] Zhang Y.X. (2023). Effects of Planting Methods and Organic Fertilizer Application on the Growth and Quality of Dandelion. Master’s Thesis.

[35] Jiang X.M., Zhang X., Cheng Y., Mao J.Y., Lei B., He X.X., Liu X.B., Li P.X., Pan K. (2023). Effects of light intensity on growth, accumulation of active constituents and antioxidant activities of *Taraxacum mongolicum* Hand. Mazz. J. Northeast Agric. Univ..

[36] Cao S.Y. (2021). Effects of Water Supply on the Quality and Functional Substances of Different Kinds of *Sonchus oleraceus* L.. Master’s Thesis.

[37] Anca T., Laurian V., Ilioara O., Dan C.V., Ana-Maria G., Ilioara O. (2019). *Solidago graminifolia* L. Salisb. (Asteraceae) as a valuable source of bioactive polyphenols: HPLC profile, in vitro antioxidant and antimicrobial potential. Molecules.

[38] Elżbieta S.S., Marlena D.M., Justyna C.K., Natasza C.K., Rafał R., Joanna Ł., Karolina G., Wiesława B., Janusz W. (2018). Anti-inflammatory activity and phytochemical profile of *Galinsoga parviflora* Cav. Molecules.

[39] Shen Q., Lou L.Y., Yin P., Huang R., Ye X.Q., Chen J.C. (2018). Phenolic compounds and antioxidant capacity of five pickled and dried mustard brands. Food Sci..

[40] Zhang S.J., Shang Y.H. (2024). Comparative analysis of active components and antioxidant capacity of different parts of blueberry. China Condim..

[41] Guo R.F., Deng Y.P., Huang Z.K., Lai Z.X. (2016). Bioactive substances and antioxidant activity in different-colored sprouts of cauliflower (*Brassica oleracea* var. botrytis L.). Fujian J. Agric. Sci..

[42] Vojvodić S., Božović D., Aćimović M., Gašić U., Zeković Z., Bebek Markovinović A., Bursać Kovačević D., Zlatković B., Pavlić B. (2025). A preliminary insight into under-researched plants from the Asteraceae family in the *Balkan peninsula*: Bioactive compound diversity and antioxidant potential. Plants.

[43] Dai L.P., Cai S.N., Chu D.K., Pang R., Deng J.H., Zheng X.L., Dai W. (2023). Identification of chemical constituents in *Blumea balsamifera* using UPLC-Q-Orbitrap HRMS and evaluation of their antioxidant activities. Molecules.

[44] Sun B., Di H.M., Zhang J.Q., Xia P.X., Huang W.L., Jian Y., Zhang C.L., Zhang F. (2021). Effect of light on sensory quality, health-promoting phytochemicals and antioxidant capacity in post-harvest baby mustard. Food Chem..

[45] Clegg K.M. (1956). The application of the anthrone reagent to the estimation of starch in cereals. J. Sci. Food Agric..

[46] Bradford M. (1976). A rapid and sensitive method for the quantization of microgram quantities of protein utilizing the principle of protein dye binding. Anal. Biochem..

[47] Folin O., Ciocalteu V. (1927). Tyrosine and tryptophane in proteins. J. Biol. Chem..

[48] Pirie A., Mullins M.G. (1976). Changes in anthocyanin and phenolics content of grapevine leaf and fruit tissues treated with sucrose, nitrate abscisic acid. Plant Physiol..

[49] Benzie I.F., Strain J.J. (1996). The ferric reducing ability of plasma (FRAP) as a measure of ‘antioxidant power’: The FRAP assay. Anal. Biochem..

[50] Re R., Pellegrini N., Proteggente A., Pannala A., Yang M., Rice-Evans C. (1999). Antioxidant activity applying an improved ABTS radical cation decolorization assay. Free Radic. Biol. Med..

