# Supplementary material for: Artificial Domestication Enhances Bioactive Profiles and Antioxidant Capacity in Two Wild Asteraceae Plants
Source: Plants (Basel). 2025 Dec 1;14(23):3662. doi: 10.3390/plants14233662 (PMC12694336; doi:10.3390/plants14233662)
Supplement: Supplementary file 1 [file plants-14-03662-s001.zip › plants-3976024-supplementary.pdf]

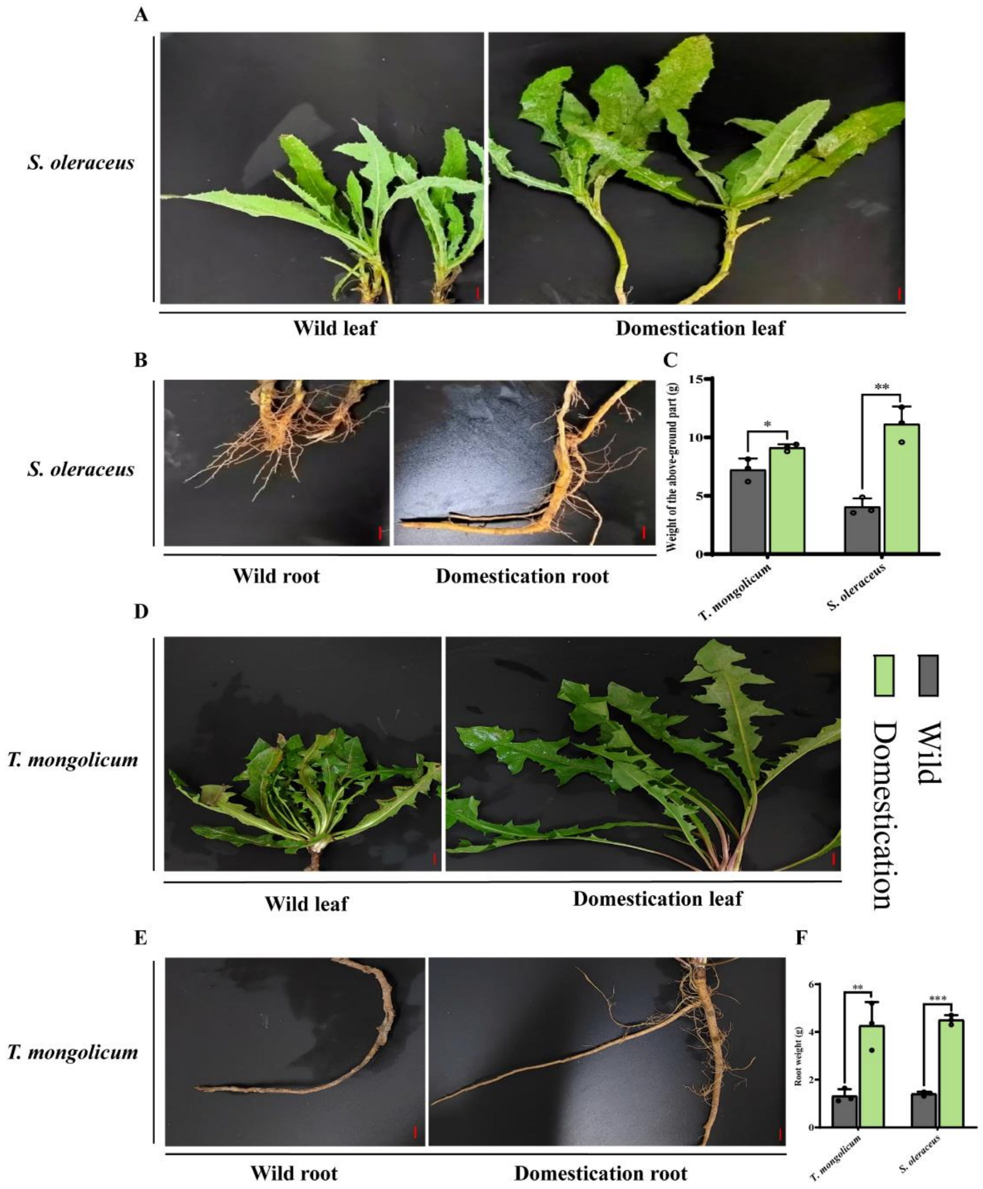

Figure S1 Detailed phenotypes of leaves and roots in two kinds of wild and domesticated vegetables. “_”, this scale shows a length of 1 cm.
